# Necrosis in the flexor hallucis longus muscle after subclinical leg compartment syndrome and tibial fracture: A case report

**DOI:** 10.1016/j.ijscr.2020.06.059

**Published:** 2020-06-17

**Authors:** R. Andri Primadhi, Gibran T. Alpharian, Renaldi Prasetia

**Affiliations:** Department of Orthopaedics and Traumatology, Universitas Padjadjaran Medical School/Hasan Sadikin Hospital, Jalan Pasteur 38, Bandung, 40161, Indonesia

**Keywords:** FHL, Necrosis, Contracture, Compartment syndrome, Ankle fusion

## Abstract

**Introduction:**

Compartment syndrome is a condition in which an increased pressure within one compartment results in decreased blood flow, preventing nutrition and blood supply from reaching nerve and muscle cells. Ongoing physiological cascades can progress to local and systemic manifestations. This article presents a case of subclinical compartment syndrome resulting in muscle necrosis and contracture. More specifically, this article highlights the presentation of a complicated and neglected case of subclinical compartment syndrome with a subsequent fixed equinus deformity and its management.

**Presentation of case:**

A 15-year-old high school student sustained a proximal tibia fracture during a motor vehicle accident. The compartment was not recognized until a period of follow-up. The patient later presented to our clinic with ankle equinus deformity. Further exploration found the contracture and necrosis of flexor hallucis longus (FHL) muscle. We excised the necrotic wound and performed an ankle fusion.

**Discussion:**

The patient had developed subclinical localized deep posterior compartment syndrome in the distal portion of the FHL muscle. As only a small portion of the muscle was involved, there was no retraction of the main muscle belly; however, the necrotic part can become fibrotic and adhere to the surrounding tissues. While subclinical compartment syndrome is not uncommon, the finding of isolated FHL muscle necrosis is rare. Thus, the management is still debatable.

**Conclusion:**

This article emphasized that neglecting compartment syndrome may result in undesired complications, which emphasizes the importance of an early diagnosis and treatment.

## Highlights

• Undetected compartment syndrome can cause disabling complications.
• In untreated compartment syndrome, nerve and vascular injury results in motor weakness, contractures, or muscle necrosis.
• Thorough examination must be done to identify the affected stuctures.
• The treatment should be based on functional outcomes, e.g., a stable foot for pain-free, plantigrade gait.
• Surgical therapy is indicated if the ankle joint cannot be restored to a functional position with conservative methods.

## 1 Introduction

Compartment syndrome can develop after a fracture. The incidence of acute compartment syndrome with tibia fractures ranges from 2% to 9% [1]. An elevated pressure causes a decrease in capillary blood flow due to a decrease in the pressure gradient at the microcirculation level. Delayed or missed diagnosis of compartment syndrome may result in nerve injury, muscle necrosis, and loss of function [2].

Although advancements have been made in orthopaedics diagnostics, occasionally there is a delay in the diagnosis of compartment syndrome. Different approaches are needed for the diagnosis of this condition, as multiple irreversible pathologies have occurred. The goal of treatment is to restore function by treating mechanical and structural impairments.

This work has been reported in line with the SCARE 2018 criteria [3].

## 2 Case presentation

A 15-year-old high school student sustained a proximal tibia fracture during a motor vehicle accident. He was brought to a local hospital and underwent open reduction and internal fixation one day later. Compartment syndrome was not observed at the time. The surgery and subsequent wound care were performed uneventfully.

**Fig. 1:**
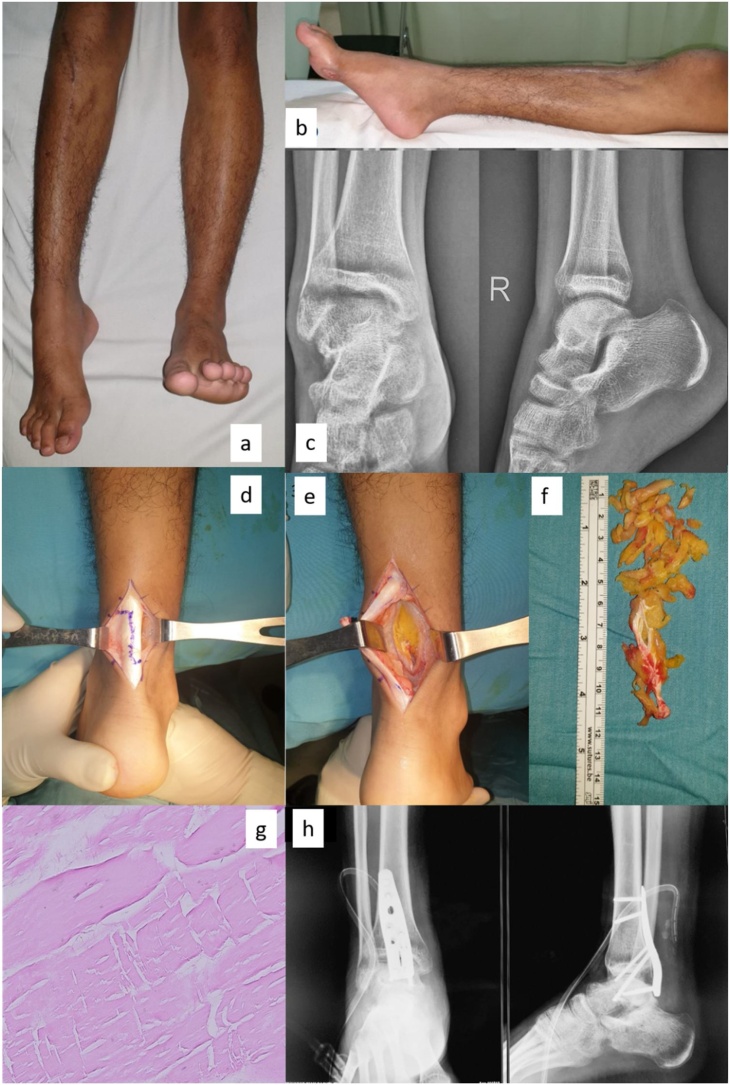
Pre-, intra-, and post-operative findings. (a, b) Frontal and lateral views of legs showing a fixed ankle equinus deformity. (c) Ankle plantarflexion showed on x-ray anteroposterior and lateral views, denoting no bony derangement. (d) Z-lengthening of Achilles tendon for addressing Achilles tendon contracture. (e) Intraoperative finding of deep posterior compartment, showing necrotic FHL muscle. (f) Necrotic FHL muscle after excision with ruler as a scale. (g) A 40x haematoxylin-eosin stained histopathological examination revealed hyalinized fibrocollagen, lymphocytic inflammatory cells infiltration, vascular congestion, without signs of malignancy. (h) Ankle arthrodesis state using posterior plate seen on immediate post-operative x-ray.

The patient presented three months later to our foot and ankle clinic with an obvious fixed equinus deformity of the right ankle and foot with a “soft stop” (Fig. 1a and b). The physical examination revealed an Achilles tendon contracture, with signs of peroneal nerve palsy. It was difficult to evaluate his ability to dorsiflex the foot because of the Achilles tendon contracture, but we found weakness in the toe extensors. There was decreased sensation in the top of the foot. The dorsalis pedis pulse and capillary refill time were comparable to those on the contralateral side. No lacerations were found, and no prior injuries to the ankle were found.

A conventional ankle radiograph was obtained and demonstrated a plantarflexed foot (Fig. 1c).

The differential diagnosis for this problem can include fixed equinus due to Achilles tendon contracture and contractures in other plantarflexor muscles due to weakness in the foot extensors or prolonged immobilization and a lack of rehabilitation. We excluded arthrofibrosis and bony ankylosis because there was a “soft stop” instead of a “hard stop” in the dorsiflexion examination.

Considering the extensor muscles as well as the antagonist tendons were weak, we decided to perform deformity correction through Achilles tendon lengthening followed by ankle fusion using the same posterior trans-Achilles approach (Fig. 1d). The procedure was performed by the first author (RAP). The goal was to restore stability in the foot to enable the patient to walk with a normal, plantigrade gait pattern.

Intra-operatively, after Achilles tendon lengthening, we found that the ankle could still not be reduced into the plantigrade position. Further exploration revealed a contracture and necrosis of the flexor hallucis longus (FHL) tendon (Fig. 1e and f). We excised all the necrotic muscles and tendons. The excised tissue was sent for a histopathological examination (Fig. 1g). Ankle fusion was performed using a posterior ankle fusion plate (Fig. 1h).

The wound healed uneventfully after the operation. The rehabilitation protocol was as follows: 3 weeks of non-weight-bearing, followed by gradually increased partial weight-bearing until 8 weeks, and full weight-bearing as tolerated starting at 8 weeks post-operatively.

The patient reported no significant problems after the last follow-up visit. The adherence to post-operative rehabilitation protocol was satisfactory.

## 3 Discussion

Skeletal muscle is the dominant type of tissue in the calf and is most vulnerable to ischaemia. An increase in the compartment pressure induces compartment syndrome, and as the tissue pressure exceeds the venous pressure, blood outflow becomes impaired. This series of events eventually leads to ischaemic necrosis of muscles and nerves [[4], [5], [6]].

Young people, especially men, have a high incidence of acute compartment syndrome due to their relatively large muscle volumes, and the compartment size does not change after growth is complete. Thus, young men may have less space for swelling of the muscle after exercise [7].

This patient showed no clinical evidence of compartment syndrome of the leg. The intraoperative findings, however, demonstrated localized necrosis of the distal part of the FHL muscle, as confirmed by a histological examination. Proximal to this area, the muscle was visually normal. The best explanation for these findings is that the patient had developed subclinical localized deep posterior compartment syndrome in the distal portion of the FHL muscle. As only a small portion of the muscle was involved, there was no retraction of the main muscle belly; however, the necrotic part can become fibrotic and adhere to the surrounding tissues [8]. This condition also co-occurred with drop foot due to subtle anterior compartment syndrome of the leg, resulting from a proximal tibial fracture or fixation with a plate on the lateral side. Iatrogenic injury to the deep peroneal nerve in the distal portion of the leg may occur during surgical manoeuvres performed for proximal tibial fractures, including incisions, blunt dissection to the plate, drilling, and the insertion of a screw [9].

The posterior trans-Achilles approach was chosen because both Achilles tendon lengthening and ankle fusion can be completed using this approach and the same position. Thus, the risk of morbidity due to an additional incision is reduced. We performed Z-lengthening for the Achilles tendon because of the substantial state of contracture [10]. A posterior ankle fusion plate (OsteoMed, USA) was applied to preserve the subtalar joint.

Partial excision or transfer of the distal muscle belly and tendon FHL result in decreased flexion power at the IP joint, which can be demonstrated by a decreased distal phalangeal pulp pressure. However, this condition does not significantly affect individuals’ gait, as their level of function typically remains high [11].

The limitation of this report is not to compare different treatment choices, for example with non-operative management, or FHL release followed with orthosis without an ankle fusion procedure. This report added some important points to existing literature, such as the histological finding of the contractured necrotic muscle resulting from subclinical compartment syndrome, and the importance not to miss this condition in similar clinical settings, especially in developing countries when patient’s adherence is not dependable.

## 4 Conclusion

Neglecting compartment syndrome may result in undesired complications, which emphasizes the importance of an early diagnosis and treatment. FHL muscle necrosis is one of the sources of equinus deformity after subclinical compartment syndrome. Therefore, the thorough examination is important for managing this complication.

## Declaration of Competing Interest

The authors declare no conflict of interest in this case report.

## Sources of funding

There are no funding source for this case report.

## Ethical approval

This study is exempted from ethical approval in Hasan Sadikin Hospital, Bandung, Indonesia.

## Consent

Written informed consent was obtained from the patient for publication of this case report and accompanying images. A copy of written consent is available for review by the Editor-in-Chief of this journal on request.

## Author contribution

**Raden Andri Primadhi**: Conceptualization, Methodology, Investigation, Writing - Original Draft, Project administration. **Gibran T. Alpharian**: Investigation, Data curation, Writing - Review & Editing. **Renaldi Prasetia**: Investigation, Data curation, Writing - Review & Editing.

## Registration of research studies

Not applicable.

## Guarantor

Raden Andri Primadhi, MD

Department of Orthopaedics and Traumatology

Universitaas Padjadjan Medical School/Hasan Sadikin Hospital

Jalan Pasteur 38, Bandung

Indonesia 40161.

## Provenance and peer review

Not commissioned, externally peer-reviewed.

## Patient perspective

“I think I should have visited the clinic for follow-up visits regularly. At first, I thought it was just a delayed functional improvement, not a serious complication like this. Now, I am so thankful that I can return to my activities independently, even though I have a limited range of motion.”
